# The Effect of Scaphoid Morphology After Surgical Fixation of Scaphoid Nonunions on Long-term Functional Outcomes and Scaphoid Union Rates

**DOI:** 10.1055/a-2745-8232

**Published:** 2025-12-03

**Authors:** Isabelle E.L. Tottenham, Tiffany K. Coromina, Gurpreet Dhaliwal, Eric C. Sayre, Yibo Li, Neil J. White

**Affiliations:** 1 Faculty of Medicine & Dentistry, University of Alberta, Edmonton, AB, Canada; 2 Department of Orthopedics, Makati Medical Center, Manila, Philippines; 3 Department of Surgery, Cumming School of Medicine, University of Calgary, Calgary, AB, Canada; 4 British Columbia Centre on Substance Use, Vancouver, BC, Canada; 5 Western Hand & Upper Limb Facility, Sturgeon Hospital, Edmonton, AB, Canada

**Keywords:** scaphoid, nonunion, carpal injury

## Abstract

**Background:**

Scaphoid nonunion can progress to wrist pain, stiffness and an established pattern of wrist arthritis. The effect of scaphoid malunion on clinical outcomes is largely unknown. In this study, the effect of scaphoid morphology on union rates and clinical outcomes is explored using a prospectively collected database of surgeries.

**Purpose:**

The primary aim of the study is to understand the effect of initial post-surgical scaphoid morphology on union. The secondary aim examines the effect of final scaphoid morphology on long-term functional outcomes among patients that underwent surgical fixation for scaphoid nonunion and ultimately went on to union.

**Materials and Methods:**

84 participants were included in this study. 74 (88.1%) nonunions went to union, and 10 (11.9%) remained persistent nonunions after surgery.

**Results:**

After nonunion surgery, the initial post-operative height-to-length (H/L) ratio was predictive of scaphoid union. For every 0.1 increase in H/L ratio, odds of union were reduced by 53.7%. Of the patients who progressed to union, there was a significant relationship ( *p* =0.019) between initial post-operative H/L ratio and final DASH scores. It was found that there was a 35.9% increase in DASH score for every 0.1 increase in H/L ratio. Similarly, there was a significant relationship ( *p* =0.013) between final post-operative H/L ratio and final DASH scores where an increase of 0.l in H/L ratio translated to a 33.5% increase in DASH.

**Conclusion:**

The authors propose that the restoration of initial post-operative H/L ratio can predict odds of union after surgical fixation of an established scaphoid nonunion. Improved morphology at union as measured by H/L ratio also demonstrated improved functional outcomes, such as DASH score.

## Background

The scaphoid is the most fractured carpal bone and has the highest rate of nonunion among carpal bone fractures at 15.5%. 1 2 Scaphoid nonunion can progress to wrist pain, stiffness, and an established pattern of wrist arthritis known as scaphoid nonunion advance collapse (SNAC). 3 Risk factors for the development of a scaphoid nonunion can include time to surgery, initial fracture displacement, and fracture location. 4 Scaphoid fractures, in both the acute and chronic setting, can develop a characteristic deformity known as a “humpback” and the degree of deformity in the scaphoid has been demonstrated to strongly correlate with dorsal intercalated segment instability (DISI). 5 This DISI instability pattern has been implicated in the development of wrist pain, impaired range of motion, and contribution to the development wrist arthritis. 6 Correction of a humpback deformity requires bone grafting. 7 As the graft required to complete the correction increases in size, there is a greater biological demand to heal the graft. The treating surgeon should be informed as to whether attempting to achieve full correction will affect chances of union, and if so, whether it is necessary to achieve the full correction or instead “settle” for undercorrection in favor of union.

The effect of scaphoid malunion on clinical outcomes is largely unknown. There are a limited number of retrospective studies with conflicting results that assess the value of scaphoid morphology on the functional outcomes of postoperative patients. Amadio et al retrospectively analyzed 46 scaphoid fractures 6 months after union. 8 They found that malunion with a humpback deformity was progressively associated with poor clinical and radiographic results. Megerle et al reported on 65 retrospective cases of scaphoid fractures. 9 Radiolunate (RL) angle correlated significantly with functional patient outcomes such as range of motion, grip strength, and pain. Seltser et al reported on the long-term outcomes of 22 patients with malunited scaphoid fractures. 10 They report that 45% of the patients demonstrated radiographic findings of early arthritis.

In this study, the effect of scaphoid morphology on union rates and clinical outcomes is explored using a prospectively collected database of surgeries. The primary aim of the study is to understand the effect of initial post-surgical scaphoid morphology on union. The secondary aim examines the effect of final scaphoid morphology on long-term functional outcomes among patients who underwent surgical fixation for scaphoid nonunion and ultimately went on to union.

## Methods

### Inclusion and Exclusion Criteria

This study population originates from a subset of the SNAPU study conducted by the senior author. 11 The SNAPU study looked at the effect of low intensity pulsed ultrasound on scaphoid nonunion. The SNAPU inclusion criteria included patients who had a scaphoid fracture older than 3 months with at least one feature of nonunion (collapse/humpback deformity, sclerosis, or cystic changes) and consented to surgery for nonunion. The SNAPU exclusion criteria included subjects who had an ipsilateral concomitant fracture, subjects with an open or pathological fracture, and subjects who were actively receiving treatment for a rheumatological or arthritic condition with biologic medication.

The current study sourced participants from the SNAPU study. This study included those patients with waist fractures with established nonunion and humpback deformity requiring correction. Patients with distal or proximal third fractures were excluded from the current study. Patients without humpback deformity (sclerosis or cystic changes) were also excluded from the current study.

### Patients

**Fig. 1 CONSORT (Consolidated Standards of Reporting Trials) diagram of study population.**
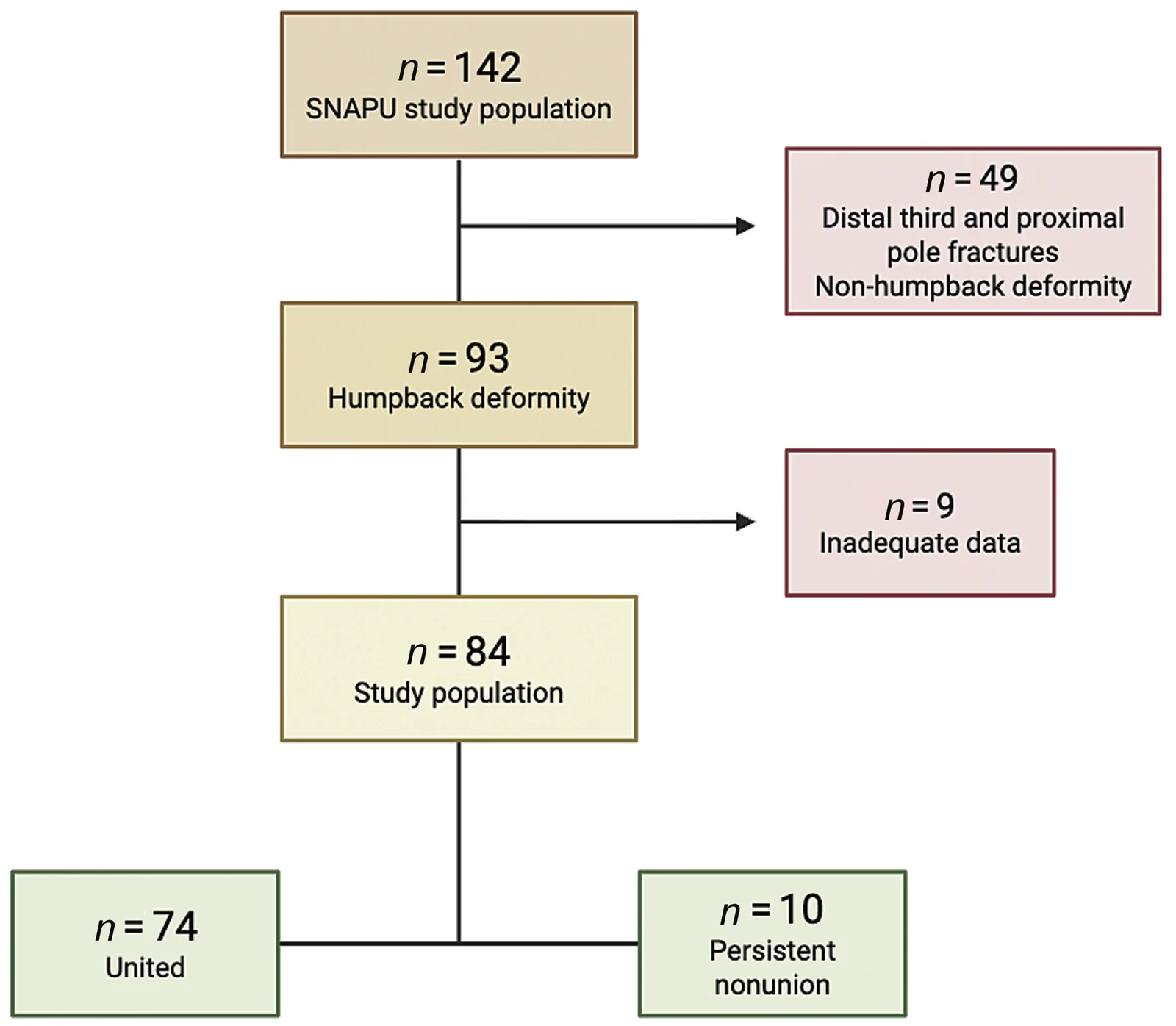
Visual representation of inclusion and exclusion criteria from the SNAPU study population dataset. A total of 84 patients were included in the study to assess the relationship between postoperative measurements (H/L, ISA, RL, SL) and union. Data from patients who went to union ( *n* = 74) were used to determine the relationship between postoperative measurements and the preoperative to postoperative change in functional outcomes (DASH, grip strength, VAS, wrist extension, wrist flexion).

Fig. 1 shows the study population. The SNAPU trial comprised of 142 participants. In this study, the fracture pattern of interest was specific to those with waist fractures with humpback deformity. From the SNAPU trial population, 90 waist fractures and 3 atypical fractures with humpback deformity were selected. All underwent surgical correction of deformity with bone grafting. Nine participants were excluded due to missing imaging data required for measurements in this study. In total, 84 participants were included in this study. After surgery 74 (88.1%) nonunions went to union, and 10 (11.9%) remained persistent nonunions.

### Evaluation

Preoperative and postoperative functional assessments were collected. The assessments comprised of the Disabilities of the Arm, Shoulder, and Hand (DASH) score, visual analog scale (VAS), grip strength, and range of motion (ROM) for flexion and extension. Patients were followed at regular intervals with serial CT scans and radiographs starting at 8 weeks postoperatively. For the purposes of this study, radiographic data were collected at two time points: (1) initial postoperative and (2) final follow-up.

CT scans provide the best bony detail and resolution of the scaphoid. 3 12 13 Height-to-length (H/L) ratio and intrascaphoid angle (ISA) were obtained from postoperative CT scans. The H/L ratio has been found to be the most accurate method of assessing humpback deformity. 9 13 The ISA measured on CT has also been shown to be a good determinant of clinical outcomes in scaphoid nonunions. 8 The radiolunate (RL) angle and the scapholunate (SL) angle were measured radiographs. 9 The RL angle is a useful indicator for carpal instability and was noted to have a relationship with the functional outcomes of patients. 9 Union was defined as greater than 50% trabecular bridging on CT scans or evidence of healing on serial X-rays. 14 Measurements and union status were reviewed by an adjudication committee comprised of three independent surgeons.

### Statistics

Logistic regression modeling was used to evaluate the effect of initial postoperative scaphoid morphology on the odds of union. The odds ratio (OR) represented the multiplicative effect of a per-unit increase in the explanatory variable on the odds of the outcome. For each model we also computed area under the receiver operating characteristic curve (AUC). Linear regression models were used to evaluate the effect of final scaphoid morphology on long-term functional outcomes (DASH, VAS, grip strength, extension, and flexion). Significance was set at *p* < 0.05. Statistical analysis of the descriptives ( Tables 1 and 2 ) was performed using a Wilcoxon signed-rank test for the Δ values. In these analyses, * *p* < 0.05, ** *p* < 0.01, and *** *p* < 0.001.

**Table 1:** Mean radiographic measurements in patients who progressed to scaphoid union

Postoperative measurements		*n*	Mean ± SD	Range	*P* value
H/L ratio	Initial Final Δ	71 63 61	0.67 ± 0.12 0.71 ± 0.11 0.02 ± 0.10	0.06–0.96 0.36–1.00 −0.38–0.32	- - a *0.027*
ISA	Initial Final Δ	71 63 61	41.85 ± 20.51 46.90 ± 22.17 3.90 ± 16.67	7.69–86.30 1.40–94.70 −26.00–52.60	- - 0.120
RL	Initial Final Δ	72 73 71	16.82 ± 10.59 12.22 ± 9.73 −4.35 ± 9.28	1.20–55.40 0.00–39.13 −31.00–27.50	- - c *<* *0.001*
SL	Initial Final Δ	72 73 71	50.26 ± 10.37 52.82 ± 11.02 3.01 ± 11.55	20.50–78.10 24.50–78.30 −20.50–57.80	- - a *0.041*

Abbreviations: H/L, height-to-length; ISA, intrascaphoid angle; RL, radiolunate angle; SD, standard deviation; SL, scapholunate angle.

Notes: Initial and final postoperative measurements were collected for H/L ratio, ISA, RL, and SL. Data are given as count, mean ± SD, and minimum – maximum (range). Δ indicates the change between preoperative baseline and final postoperative measurements. Wilcoxon signed-rank tests were calculated for Δ.

a *p* < 0.05.

b *p* < 0.01.

c *p* < 0.001.

**Table 2:** Mean functional outcomes in patients who progressed to scaphoid union

Functional outcome measurements		*n*	Mean ± SD	Range	*P* value
DASH	Preoperative baseline Final postoperative Δ	72 67 66	30.14 ± 18.78 9.43 ± 13.01 −20.38 ± 16.11	0.00–85.00 0.00–81.87 −58.33–4.16	- - c *<* *0.001*
Grip	Preoperative baseline Final postoperative Δ	69 65 61	32.52 ± 13.52 39.81 ± 13.73 8.53 ± 11.77	0.34–80.00 3.34–72.00 −8.00–40.80	- - c *<* *0.001*
VAS	Preoperative baseline Final postoperative Δ	72 66 65	28.224.90 10.18 ± 15.12 −17.43 ± 24.38	0.00–83.33 0.00–76.67 −69.33–38.34	- - c *<* *0.001*
Wrist extension (°)	Preoperative baseline Final postoperative Δ	71 65 63	50.10 ± 17.52 61.54 ± 14.81 13.27 ± 19.71	5.00–88.00 20.00–110.00 −18.00–75.00	- - c *<* *0.001*
Wrist flexion (°)	Preoperative baseline Final postoperative Δ	71 65 63	63.10 ± 13.58 57.69 ± 13.01 −4.46 ± 17.06	38.00–90.00 15.00–85.00 −50.00–33.00	- - a *0.022*

Abbreviations: DASH, Disabilities of the Arm, Shoulder and Hand; SD, standard deviation; VAS, visual analog scale.

Notes: Preoperative baseline and final postoperative measurements were collected for DASH, grip strength, VAS, wrist extension, and wrist flexion. Data are given as count, mean ± SD, and minimum – maximum (range). Δ indicates the change between preoperative baseline and final postoperative measurements. Wilcoxon signed-rank tests were calculated for Δ.

a *p* < 0.05

b *p* < 0.01

c *p* < 0.001.

## Results

A total of 84 operated scaphoid nonunions were included in this study ( Table 3 ). Of the included patients, 75 were male and 9 were female. The average age was 29.90 ± 9.66 years old (range: 17.00–61.00 years). After nonunion surgery, the initial postoperative H/L ratio was predictive of scaphoid union. A normal H/L ratio was defined as ranging from 0.54 to 0.69. 13 15 The mean of the initial postoperative H/L ratio was 0.68 ± 0.12 ( *n* = 81). Area under the ROC curve (AUC) allows for the evaluation of the accuracy of a predictor variable of interest. An AUC of 1 indicates a perfectly accurate test, and an AUC of 0.5 suggests no discrimination. An AUC of 0.7 to 0.8 is considered acceptable. The AUC for H/L ratio demonstrated acceptable predictive utility (0.745). Initial postoperative H/L ratio had an odds ratio of 0.463 in predicting union ( *p* = 0.018). A larger H/L ratio suggest more residual deformity after surgery. For every 0.1 increase in H/L ratio, odds of union were reduced by 53.7%. ISA, RL, and SL did not show statistically significant predictive utility ( Table 4 ).

**Table 3:** Study population demographics

Characteristic	Value (%)
Age	29.90 ± 9.66 years Range: 17.00–61.00 years
Sex Female Male	9 (10.7) 75 (89.3)
Hand dominance Right Left	75 9
Fracture side Right Left	52 32
Union at final follow-up United Nonunited	74 (88.1) 10 (11.9)

Note: Demographic information of patients included in the study ( *n* = 84). Data are given as count with the percentage in parentheses (%) or as the mean ± SD.

**Table 4:** Initial postoperative H/L ratio predicts union

	Low OR	OR	Upper OR	*P* value	Std. error	Utility (AUC)
**H/L ratio/0.1**	**0.245**	**0.463**	**0.876**	**0.018**	**0.325**	**0.745**
ISA/10	0.593	0.818	1.129	0.221	0.164	0.625
RL/10	0.575	1.105	2.123	0.764	0.333	0.544
SL/10	0.527	1.015	1.954	0.964	0.334	0.516

Abbreviations: AUC, area under the curve; H/L, height-to-length; ISA, intrascaphoid angle; OR, odds ratio; RL, radiolunate angle; SL, scapholunate angle; Std. error, standard error.

Notes: Logistic regression model of initial postoperative measurements (H/L, ISA, RL, SL) and scaphoid union. Data are presented as the spectrum of odds ratios with standard error and utility. Utility is determined by area under the curve (AUC).

a *p* < 0.05.

Table 1 reports the mean postoperative measurements of scaphoid union: H/L ratio, ISA, RL, and SL. Initial H/L ratio was 0.67 ± 0.12 ( *n* = 71) and final H/L ratio was 0.71 ± 0.11 ( *n* = 63). Initial ISA was 41.85 ± 20.51 ( *n* = 71) and final ISA was 46.90 ± 22.17 ( *n* = 63). Initial RL was 16.82 ± 10.59 ( *n* = 72) and final RL was 12.22 ± 9.73. Initial SL was 50.26 ± 10.37 ( *n* = 72) and final SL was 52.82 ± 11.02 ( *n* = 73).

Table 2 summarizes the mean measurements of functional outcomes in the study. Baseline DASH was 30.14 ± 18.78 ( *n* = 72) and final DASH was 9.43 ± 13.01 ( *n* = 67). Baseline grip was 32.52 ± 13.52 ( *n* = 69) and final grip was 39.81 ± 13.73 ( *n* = 65). Baseline VAS was 28.28 ± 24.90 ( *n* = 72) and final VAS was 10.18 ± 15.12 ( *n* = 66). Baseline extension was 50.10 ± 17.52 ( *n* = 71) and final extension was 61.54 ± 14.81 ( *n* = 65). Baseline flexion was 63.10 ± 13.58 ( *n* = 71) and final flexion was 57.69 ± 13.01 ( *n* = 65). All Δ functional outcomes measurements were significant.

Table 5 highlights the relationship between initial and final postoperative measurements and the follow-up DASH score. Of the patients who progressed to union ( *n* = 74), there was a significant relationship ( *p* = 0.019) between initial postoperative H/L ratio and final DASH scores. For every 0.1 increase in initial postoperative H/L ratio, there is a 0.307 increase in log of final follow-up DASH score. This translates to a 35.9% increase in DASH score for every 0.1 increase in H/L ratio. Similarly, there was a significant relationship ( *p* = 0.013) between final postoperative H/L ratio and final DASH scores in those that progressed to union. For every 0.1 increase in final postoperative H/L ratio, there is a 0.289 increase in log of final follow-up DASH score. This translates to a 33.5% increase in DASH score for every 0.1 increase in H/L ratio. Initial and final postoperative measurements of ISA, SL, and RL showed no statistical significance in relation to DASH scores.

**Table 5:** H/L ratio correlates with follow-up in DASH score

	Lower CL	Estimate	Upper CL	*P* value	Std. error
Initial postoperative measurements					
**H/L ratio/0.1**	**0.051**	**0.307**	**0.563**	**0.019**	**0.128**
ISA/10	−0.036	0.128	0.293	0.125	0.082
RL/10	−0.238	0.096	0.429	0.568	0.167
SL/10	−0.275	0.045	0.365	0.779	0.160
Final postoperative measurements					
**H/L ratio/0.1**	**0.062**	**0.289**	**0.516**	**0.013**	**0.113**
ISA/10	−0.058	0.107	0.273	0.200	0.083
RL/10	−0.327	0.020	0.368	0.907	0.174
SL/10	−0.208	0.091	0.390	0.546	0.150

Abbreviations: CL, confidence limit; DASH, Disabilities of the Arm, Shoulder and Hand; H/L, height-to-length; ISA, intrascaphoid angle; RL, radiolunate angle; SL, scapholunate angle; Std. error, standard error.

Notes: Linear regression model of postoperative measurements with the follow-up DASH score. Data are presented as lower and upper confidence limits, mean CL (estimate), and standard error.

a *p* < 0.05.

Table 6 shows the relationship between initial and final postoperative measurements and the follow-up wrist extension range of motion (ROM). Of the patients who progressed to union ( *n* = 74), there was a significant relationship between initial H/L ratio and extension ( *p* = 0.008) and final H/L ratio and extension ( *p* = 0.001). In the cases of both initial and final H/L ratio, for every 0.1 increase there is a 0.091 decrease in wrist extension ROM. In other words, for every 0.1 increase in initial or final H/L ratio, there was an 8.7% reduction in extension.

**Table 6:** H/L ratio and RL correlate with extension measurements

	Lower CL	Estimate	Upper CL	*P* value	Std. error
Initial postoperative measurements					
**H/L ratio/0.1**	**−0.158**	**−0.091**	**−0.024**	**0.008**	**0.033**
ISA/10	−0.033	0.002	0.037	0.906	0.018
** RL/10**	**−0.129**	**−0.066**	**−0.003**	**0.041**	**0.032**
SL/10	−0.059	0.004	0.066	0.903	0.031
Final postoperative measurements					
**H/L ratio/0.1**	**−0.144**	**−0.091**	**−0.038**	**0.001**	**0.026**
ISA/10	−0.038	−0.003	0.032	0.855	0.017
RL/10	−0.070	−0.002	0.067	0.962	0.034
SL/10	−0.112	−0.054	0.005	0.070	0.029

Abbreviations: CL, confidence limit; H/L, height-to-length; ISA, intrascaphoid angle; RL, radiolunate angle; SL, scapholunate angle; Std. error, standard error.

Notes: Linear regression model of postoperative measurements with follow-up extension measurements. Data are presented as lower and upper confidence limits (CL), mean CL (estimate), and standard error. RL was only significant in the initial postoperative measurements, while H/L ratio was statistically significant in both initial and final postoperative measurements.

a *p* < 0.05.

There was also a significant relationship ( *p* = 0.041) between initial RL measurement and wrist extension ROM ( Table 6 ). There is a 0.066 decrease in wrist extension ROM for every 10 unit increase in RL measurement. This means that for every 10 unit increase in RL, there was a 6.4% decrease in extension ROM.

## Discussion

The purpose of this study was to investigate the relationship between scaphoid morphology, union status, and long-term functional outcomes after surgery for scaphoid nonunion. Our findings indicate that surgical restoration of scaphoid morphology as measured by H/L ratio is associated with higher union rates and improved DASH scores in patients who progressed to union. For every 0.1 increase in the initial postoperative H/L ratio, the odds of union are reduced by 53.7%. Of the patients who progressed to union, for every 0.1 increase in initial or final postoperative H/L ratio, there was an increase in DASH score of 35.9 or 33.5%, respectively. There was also a statistically significant relationship between H/L ratio and wrist extension. Of the patients who progressed to union, for every 0.1 increase in H/L ratio there is an 8.7% reduction in wrist extension. Improving H/L ratio appears to be better for both union and function. However, this requires a larger graft and may be asking more out of the patient's biology to achieve union. The results of this study suggest the importance of restoring normal or near-normal scaphoid morphology, as measured by H/L ratio, in surgical management of established scaphoid nonunions.

Although our data suggests that correction of scaphoid morphology has positive benefits in both union rates and functional outcomes, there are significant challenges in quantifying scaphoid deformity on radiographic and CT imaging. 16 Although height to length ratio of the scaphoid has been shown to have the highest inter-rater reliability, 13 there remains a lack of consensus regarding the normal range of H/L ratio. 15 Additionally, the non-united scaphoid often has dorsal osteophytes obfuscating the true measurement of both height and length. It is unclear whether altering the axis of a CT scan from anatomic wrist to anatomic scaphoid can reliably quantify a scaphoid deformity. Cheema et al demonstrated that reformatting CT scans along the axis of the scaphoid did not provide improved reliability in measurements of scaphoid deformity or displacement. 17 Conversely, Lee et al argue that in measuring H/L ratio, scaphoid axis reformatting of the CT scan shows superior reliability compared to a carpal axis CT. 18 Assessing the scaphoid with three-dimensional CT imaging may provide a better understanding for surgical approach. 15 However, a standardized method to measure H/L ratio and understanding its predictive value is not clear.

It was striking that H/L ratio was the only measure correlating to both union and patient outcomes. There are contrasting reports regarding the use of H/L ratio to predict functional outcome. Forward et al reported no correlation between outcome measures (ROM, grip strength, DASH) and the measures of malunion (H/L ratio, dorsal cortical angle, and ISA) after 1 year in patients with scaphoid waist fracture. 19 It is also reported that preoperative H/L ratio does not predict union status or functional outcome. 20 This highlights a limitation in our study in that we were unable to assess preoperative H/L ratio as the SNAPU study data did not have consistent preoperative CT imaging. Although the postoperative values are of more importance, we are unable to calculate the degree of correction in these patients. Interestingly, there was very little loss of correction between initial postoperative and final postoperative values. The senior authors had expected more collapse over the average 64 weeks (range: 24–116 weeks) between initial and final postoperative CT. To our knowledge, serial CT measurements such as this have not been documented.

The current study highlights that the appropriate correction seems to predict outcomes. Future studies could be directed toward looking at the size of correction as it pertains to union and functional studies. There are other noteworthy limitations in this study. The population data collected in this study were repurposed from a previous study and analyzed retrospectively. Ideally, the data should have come from a multi-center prospective study, designed specifically to address the gaps in knowledge surrounding scaphoid morphology. Unfortunately, there were limited preoperative CT scans available for measurement and therefore the degree of correction in scaphoid morphology from pre- to postoperative could not be assessed. Additionally, the measurements used in this study are difficult. The measurements were completed by three independent surgeons, minimizing discrepancies in calculations. However, there is still the possibility of human error associated with measurements.

## Conclusion

Restoration of initial postoperative H/L ratio can predict odds of union after surgical fixation of an established scaphoid nonunion. Improved morphology at union as measured by H/L ratio also demonstrated improved functional outcomes in terms of DASH scores and wrist extension ROM. ISA, RL, and SL did not demonstrate any significant correlations to union rates and functional outcomes in this study. This study highlights the importance of restoring scaphoid length.
